# Bacillus Decalvans

**Published:** 1886-04

**Authors:** 


### BACILLUS DECALVANS.

Prof. Von Schlen has recently discovered the bacillus of alopecia areata; has cultivated it and has succeeded in producing areas of baldness in the lower animals, by inoculating with the culture fluid. Now what is wanted is somebody to learn how to prevent baldness, since the Professor has discovered how to produce it. Immortality awaits the man who will discover a microbe that will make the hairs grow instead of one that will prevent it.

---

Speaking of the coming meeting of the Texas State Medical Association, which is to be held in Dallas this month, the *Mississippi Valley Medical Monthly* (*Memphis*) says:

For the past few years this Society has developed more than an average interest in the minds of the profession throughout the State, and now stands one of the most prosperous medical associations in the country. If we are permitted to judge of the causes that have operated to bring about such results, we will unhesitatingly, mention as a prime factor, *Journalism.* We have noticed that our local and State societies, whether they owe their prosperity to this source or not, move in that direction *pari passu* with a live home medical periodical. Texas has two good medical journals, with DANIEL pushing one, and Brooks behind the other.

---

Dr. Holt has withdrawn his resignation as President of the Louisiana State Board of Health.

---

Dr. Flint bequeathed his library to the New York Academy of Medicine.

---

A good country location for an educated physician of experience, for sale. Address Dr. G. W. Christian, Burnett, Texas.

